# Relative bioavailability of dietary iron sources in growing Japanese quail: Multi-endpoint dose-response and Random Forest analyses

**DOI:** 10.1016/j.psj.2026.107754

**Published:** 2026-09-13

**Authors:** Mahmoud Ghazaghi, Meysam Pourtaheri, Farzad Bagherzadeh-Kasmani, Mehravar Mehri, Hassan Saleh, Morteza Asghari-Moghadam, Mehran Mehri

**Affiliations:** a Department of Animal Sciences, College of Agriculture, University of Zabol, Zabol, 98613-35856, Iran; b Faculty of Agriculture, University of Torbat Heydarieh, Torbat Heydarieh, Iran

**Keywords:** Bioavailability, Chelated-Fe, Exponential regression, Nanoparticles, Random forest, Slope-ratio assay

## Abstract

The present study evaluated the relative bioavailability of Fe supplied as Fe sulfate (FeSO₄), chelated-Fe, nano-Fe oxide, and Fe oxide in growing Japanese quail. A total of 1,200 quails (21 d of age) were allocated to 20 treatments consisting of four Fe sources and five dietary Fe concentrations (30, 60, 90, 120, and 150 mg Fe/kg diet), with five replicate pens of 12 birds each. Growth performance, carcass traits, reproductive organ development, bone characteristics, mineral deposition, and fecal Fe excretion were determined. Relative bioavailability values (RBV) were estimated using linear slope-ratio assays or nonlinear exponential regression models according to the statistical validity of each response criterion. Random Forest analysis was additionally used to identify the biological traits most informative for predicting dietary Fe concentration. Fe source and supplementation level significantly affected body weight gain (P = 0.023 and P < 0.001, respectively) and feed conversion ratio (P = 0.012 and P < 0.001, respectively), with a significant source × level interaction for feed conversion ratio (P = 0.014). Testis weight and tibial Fe concentration were also affected by Fe source, supplementation level, and their interaction (P < 0.05). Chelated-Fe and nano-Fe generally produced greater tibial Fe concentrations than FeSO₄ and Fe oxide, whereas Fe oxide resulted in greater fecal Fe excretion (P < 0.001). Statistical validation supported linear slope-ratio analysis for selected response criteria, including bone density, bone volume, tibial zinc, and fecal Fe, whereas relative bone weight and thigh meat yield showed nonlinear responses. Slope-ratio analysis yielded RBV estimates of 116.6 % for chelated-Fe and 75.3 % for nano-Fe for bone density, while fecal Fe-based estimates were 101.2 % and 84.8 %, respectively, compared with FeSO₄; Fe oxide showed the highest fecal Fe-based estimate (136.3 %). Nonlinear analysis produced numerically favorable RBV estimates for chelated-Fe for relative bone weight (151.2 %) and thigh meat yield (118.0 %), but the associated confidence intervals indicated substantial uncertainty. Random Forest analysis identified FeFecal, relative bone weight, fecal ash, and liver Fe as the most informative predictors of dietary Fe concentration. Overall, Fe source influenced biological responses in an endpoint-dependent manner. Chelated-Fe and nano-Fe showed favorable responses for selected criteria, but the variability and uncertainty of RBV estimates preclude concluding consistent superiority over FeSO₄ across all biological endpoints.

## Introduction

Iron (Fe) has a critical role in biological processes that occur in rapidly growing domestic birds and it is essential for the function of numerous metalloenzymes such as superoxide dismutase (Barwinska-Sendra et al., 2020) and takes part in oxygen transport and DNA synthesis (Gupta, 2014). The influence of Fe deficiency on the chick has been examined previously, with studies showing it affects gut function and leads to a decrease in villus surface area (Saiz et al., 1993). The reduction of ferric Fe to ferrous Fe at the brush border of the broiler chicken for absorption is not fully understood. However, it is known that both ferric and ferrous Fe forms can be absorbed in the body through different pathways (Ebbing et al., 2019). The absorption of Fe in the body is a complex process that involves several transporters and regulatory mechanisms (Lu et al., 2018; Tako et al., 2010). The duodenal brush border membrane is the site of Fe absorption in the small intestine (Muir et al., 1985). When Fe ions (Fe^3+^ and Fe^2+^) arrive at the duodenal brush border membrane, they are transported into the enterocyte by different transporters (Goswami et al., 2002). The reduction of ferric Fe to ferrous Fe may occur at the brush border membrane or within the enterocyte. The reduction of ferric Fe to ferrous Fe is thought to be facilitated by the action of reductases, such as duodenal cytochrome b (Lu et al., 2018).

A variety of factors affecting Fe bioavailability in poultry has been suggested including Fe status, dietary factors, chemical form, Fe resources, age, genetics, and health status. Fe absorption is higher in Fe-deficient birds compared to Fe-sufficient birds (Tako et al., 2010). Dietary fiber component, phytic acid (Biehl et al., 1997), and concentrations of other minerals like calcium could interact with Fe absorption (Chausow et al., 1988; Dasa et al., 2018). Both the source and chemical form of dietary Fe can markedly affect its availability for absorption (Dasa et al., 2018). Ferric and ferrous Fe forms have been demonstrated to be absorbed in monogastric following different pathways and different potential genetics in different age classes of the birds, which can affect Fe bioavailability (Ebbing et al., 2019). Gastrointestinal environment, such as pH and gut microbiota, and the health status of poultry can also alter Fe bioavailability. For instance, high pH of the gastrointestinal system and damaged intestinal lining can reduce Fe solubility and absorption, respectively (Beasley et al., 2020; Tako et al., 2010). Ascorbic acid, as a natural acidifier, could enhance Fe absorption through binding (chelating) ferric Fe and stabilize it in the duodenum, allowing it to be more readily absorbed by the body. On the other hand, the gut microbiota can affect Fe absorption by producing compounds that chelate Fe and make it less available for absorption. In the absence of viable intestinal microbiota, the absorption and net retention of Fe could be decreased by around 25 % (Yilmaz et al., 2018). Moreover, stressful conditions, such as heat stress or transportation stress, can disrupt normal physiological processes, including Fe metabolism (Beasley et al., 2020). Therefore, it is necessary to supply a sufficient amount of available Fe through the poultry diet. In this regard, the minimum dietary Fe requirement for broilers and growing Japanese quail are set by NRC (2026) at 80 and 120 mg of Fe per kg of diet, respectively.

During the past decades, researchers tried to increase Fe bioavailability through chelates or implementation of nanotechnology. It has been reported that chelated-Fe with proteinate (Ma et al., 2014) and amino acids (Gayathri et al., 2018; Ma et al., 2012) has a higher bioavailability than FeSO_4_ in broilers, and the use of chelates is a promising approach to improve the micronutrient bioavailability in poultry (Bai et al., 2021). Nanoparticles, with their reduced particle size and novel physical and chemical properties, are gaining increased interest for a wide range of new applications. Nanoparticles have a high surface area, allowing for better interaction in biological interfaces and improved retention time in the gut (Chen et al., 2006). The small size allows for better uptake and retention in the gut, reducing the influence of clearance mechanisms and effective delivery of compounds to targeted areas, resulting in better bioavailability. Nano-minerals, due to their reduced size, may increase mineral absorption by reducing mineral antagonisms in the intestine, leading to decreased excretion (Hillyer et al., 2001). The absorption rate of nanoparticles varies widely for different tissues and organs, with particles less than 300 nm able to reach the blood circulation, and particles smaller than 100 nm able to penetrate various tissues, including the brain, heart, kidney, spleen, liver, intestine, and stomach (Feng et al., 2009). This information suggests that mineral nanoparticles may be preferred over inorganic mineral supplements, and they have the potential to improve growth performance and physiological functions, especially when used in lower doses (Patra et al., 2020).

Iron bioavailability is commonly assessed using biological response criteria such as hemoglobin, tissue Fe concentration, and other Fe-dependent physiological responses. In comparative bioassays, a test Fe source is generally evaluated against a reference source, commonly FeSO₄, across graded dietary concentrations. The slope-ratio assay provides an established approach for estimating relative bioavailability when the dose–response relationship is linear and the required assay assumptions are satisfied (Finney, 1978; Ma et al., 2014). However, biological responses may differ among endpoints and may exhibit nonlinear dose–response patterns; therefore, evaluation of dose–response validity and response shape before estimating relative bioavailability is important. Previous poultry studies have similarly shown that estimates of Fe bioavailability can vary according to the biological response criterion used (Wang et al., 2024; Zhang et al., 2016).

Despite these approaches, it remains unclear whether the relative bioavailability of different Fe sources is consistent across biological endpoints or depends on the response criterion and dose–response pattern. Therefore, the present study addressed the question of whether chelated Fe, nano-Fe, and Fe oxide differ consistently in relative bioavailability from FeSO₄ in growing Japanese quail. We evaluated multiple biological responses across graded dietary Fe concentrations and applied linear slope-ratio or nonlinear dose–response models according to the statistical validity of each endpoint. We hypothesized that Fe-source effects would be endpoint-dependent and that model selection based on the observed dose–response relationship would provide a more appropriate assessment of relative Fe bioavailability.

## Materials and methods

### Ethical approval

All animal care and experimental procedures were conducted in accordance with institutional guidelines and were approved by the Research Animal Ethics Committee of the University of Zabol, Iran (Approval ID: AECUZ-2012-BR-2025-25).

### Experimental design and animal management

A total of 1,200 Japanese quails with average body weight of 99.3 ± 7.23 g (21 d of age) were randomly assigned to 20 experimental treatments in a completely randomized design. Treatments consisted of four dietary Fe sources and five supplementation levels arranged as a 4 × 5 factorial. Each treatment consisted of five replicate pens with 12 birds per replicate. Birds were reared from 22 to 35 d of age in floor pens (1 × 1 m) equipped with feeders and nipple drinkers and had ad libitum access to feed and water throughout the experimental period. Plastic housing and feeding facilities were used throughout the experiment to minimize potential Fe contamination from environmental and contact materials. Basal diets were formulated to meet the nutrient requirements of growing Japanese quail according to NRC (1994), except for Fe, and were provided in mash form. The basal diet contained approximately 11 mg Fe/kg before Fe supplementation (Table 1). Each Fe source was weighed separately and geometrically premixed with approximately 10-fold finely ground basal diet. The resulting premix was subsequently incorporated into the remaining basal diet and thoroughly mixed to obtain homogeneous experimental diets.

**Table 1:** Composition of basal diet.

**Item**	**Amount (%)**
**Corn, grain**	55.87
**Soybean meal**	35.0
**Corn gluten meal**	3.98
**Sunflower oil**	1.43
**Di-calcium phosphate**	0.71
**Limestone**	1.41
**Sodium bicarbonate**	0.31
**l-Lysine HCl**	0.22
**DL-Methionine**	0.34
**L-Thr**	0.1
**NaCl**	0.13
**Mineral premix**^a^	0.25
**Vitamin premix**^b^	0.25
**Nutrient specifications**	
**ME (MJ/kg)**	12.3
**CP (g/kg)**	24.5
**Linoleic acid (%)**	2.31
**CF (%)**	3.84
**Ca (%)**	0.80
**Available phosphorus (%)**	0.30
**Na (%)**	0.16
**K (%)**	0.88
**Fe (mg/kg)**	11.0
**Lys (%)**	1.35
**Met (%)**	0.71
**Met + Cys (%)**	1.08
**Thr (%)**	1.02
**DEB**^c^**(mEq/kg)**^c^	250

a Mineral premix provided per kilogram of diet: Mn (from MnSO_4_·H_2_O), 65 mg; Zn (from ZnO), 50 mg; Cu (fromCuSO_4_·5H_2_O), 8 mg; I [from Ca (IO_3_)2·H_2_O], 1.8 mg; Se, 0.30 mg; Co (from Co_2_O_3_), 0.20 mg; Mo, 0.16 mg.

b Vitamin premix provided per kilogram of diet: vitamin A (from vitamin A acetate), 11,500 IU; cholecalciferol, 2100 IU; vitamin E (from dl-α-tocopherylacetate), 22 IU; vitamin B12, 0.60 mg; riboflavin, 4.4 mg; nicotinamide, 40 mg; calcium pantothenate, 35 mg; menadione (from menadione dimethyl-pyrimidinol), 1.50 mg; folic acid, 0.80 mg; thiamine, 3 mg; pyridoxine, 10 mg; biotin, 1 mg; choline chloride, 560; choline chloride, 560 mg; Ethoxyquin,125mg.

c Dietary electrolyte balance: Na + K − Cl in mEq/kg of diet.

Supplemental Fe was added at levels calculated to achieve final dietary Fe concentrations of 30, 60, 90, 120, or 150 mg Fe/kg, rather than adding these amounts on top of the basal Fe concentration.

### Fe sources and experimental treatments

Four Fe sources were evaluated: ferrous sulfate (FeSO₄), Fe-chelate, nano-Fe, and ferric oxide (Fe₂O₃). The characteristics of the nano-Fe and Fe-chelate products, including physical form, color, particle size, purity, and other available physicochemical properties, are presented in Table 2. Nano-Fe was supplied as Fe₃O₄ powder with a particle size of 20–30 nm and a stated purity of 98 % (SkySpring Nanomaterials, Inc., Houston, TX). Fe-chelate was supplied as a white powder with a particle size of approximately 450 nm and a stated purity of 99 % (Danesh Banyan Shimi Gostar Butia Samangan Company, Kerman, Iran). FeSO₄ and Fe₂O₃ were obtained from commercial suppliers and incorporated according to their Fe content. The specific product purity and hydration state of FeSO₄ were not available in the experimental records. The Fe sources were added at calculated amounts to achieve final dietary Fe concentrations of 30, 60, 90, 120, and 150 mg Fe/kg diet. The Fe concentrations of the experimental diets, fecal ash, and tibia ash were determined by flame atomic absorption spectrophotometry following acid digestion. Measurements were performed at 248.3 nm using appropriate Fe calibration standards.

**Table 2:** Characteristics of iron oxide nano-powder (nano-Fe) and chelate iron (Fe-chelate).

Name	Iron Nanopowder^a^
**Formula**	Fe_3_O_4_
**Physical form**	Powder
**Color**	Brown
**Size**	20-30 nm
**Purity**	98 %
**Specific surface area (SSA)**	40-60 m^b^/g
**Morphology**	Spherical
**Bulk density**	0.84 g/cm^3^
**True density**	4.8-5.1 g/cm^3^
**Name**	Chelate Iron^b^
**Physical form**	Powder
**Color**	White
**Size**	450 nm
**Purity**	99 %

a SkySpring Nanomaterials, Inc. 2935 West Hollow Drive • Houston, TX • 77082.

b Danesh Banyan Shimi Gostar Butia Samangan Company, Kerman, IRAN.

### Growth performance

Birds were weighed individually at the beginning (22 d) and end (35 d) of the experiment. Body weight gain (G), feed intake (FI), and feed conversion ratio (FCR) were calculated on a replicate basis. Mortality was recorded daily and used to adjust feed conversion ratio calculations when necessary. Because growth performance records for birds receiving Fe oxide were inadvertently lost during data collection, the Fe oxide treatment was omitted from the analysis of performance traits, whereas all other measurements remained complete and were analyzed as planned.

### Carcass characteristics

At 35 d of age, three birds per replicate with body weights close to the replicate mean were selected for sampling and humanely euthanized by CO₂ inhalation in accordance with the approved animal-welfare procedures. Following defeathering and evisceration, carcass yield, dressed carcass weight, eviscerated carcass weight, breast meat yield (BMY), thigh meat yield (TMY), and the relative weights of the liver, heart, spleen, bursa, and testes were determined and expressed as percentages of live body weight. These organs were evaluated based on relative weight only and were not collected for subsequent chemical or molecular analyses.

### Bone characteristics

Following carcass evaluation, the left tibia from each sampled bird was excised and carefully cleaned of adhering soft tissues. Bone weight (g) was measured using a precision digital balance, whereas bone length (cm) was measured using a digital caliper. Bone volume (cm^3^) was determined by the water-displacement method (Zhang et al., 1997), and bone density (g/cm^3^) was calculated as the ratio of bone weight to bone volume.

### Bone and fecal ash determination

Fresh fecal samples were collected separately from each replicate during the final week of the experiment. Plastic facilities were used to minimize potential contamination of fecal samples with exogenous Fe from housing and collection materials. Fecal material was collected carefully to avoid visible contamination with feed, litter, or other foreign materials, and samples were immediately transferred to clean sample containers. Samples were dried in a forced-air oven at 105°C to constant weight, finely ground, and approximately 2 g of each sample was placed in porcelain crucibles for subsequent ash determination. Dried samples were finely ground, and approximately 2 g of each sample was placed in porcelain crucibles and incinerated in a muffle furnace at 550°C for 12 h according to AOAC (2006). After cooling in a desiccator, crucibles were weighed, and fecal ash content was expressed as a percentage of dry matter. The tibia collected during carcass evaluation were cleaned of residual tissues and defatted by petroleum ether extraction according to AOAC (2006). Briefly, bone samples were immersed in petroleum ether for 48 h to remove residual fat and subsequently air-dried. Samples were then dried at 105°C to constant weight and weighed to determine dry bone weight. Thereafter, tibiae were incinerated in a muffle furnace at 550°C for 12 h. After cooling in a desiccator, ash weight was recorded and expressed as a percentage of dry bone weight. Bone and fecal ash samples were retained for subsequent mineral analyses.

### Fe and Zn analysis

Fe and zinc concentrations in diets, fecal ash, and tibia ash samples were determined by flame atomic absorption spectrophotometry (AAS; SpectrAA 220, Varian Australia Pty Ltd., Mulgrave, Victoria, Australia) following acid digestion procedures. Briefly, ash samples were digested using concentrated nitric acid and hydrochloric acid (3:1, v/v), filtered, and diluted to a known volume with deionized water. Fe concentration was measured at a wavelength of 248.3 nm, whereas zinc concentration was determined at 213.9 nm using appropriate calibration standards. Mineral analyses were performed according to the procedures of the Association of Official Analytical Chemists (AOAC, 2006).

### Statistical analysis

Prior to statistical analysis, model assumptions were evaluated to ensure the validity of the analyses. Residual normality was assessed using the Shapiro–Wilk test together with visual inspection of normal Q–Q plots. Homogeneity of variance among treatments was examined using Levene’s test. Model residuals were plotted against fitted values to evaluate homoscedasticity and detect potential deviations from model assumptions. Potential outliers and influential observations were identified using standardized residuals, leverage values, and Cook’s distance. No violations of model assumptions or influential observations requiring exclusion were detected; therefore, all observations were retained for the final analyses. Data were analyzed as a 4 × 5 factorial arrangement using the General Linear Model (GLM) procedure. The statistical model included the fixed effects of Fe source, supplementation level, and the Fe source × supplementation level interaction. The interaction term was used to determine whether responses to increasing dietary Fe concentrations differed among Fe sources. When a significant interaction was detected (P < 0.05), simple-effect comparisons were performed to evaluate differences among Fe sources at each supplementation level and differences among supplementation levels within each Fe source. Partial eta squared (η²p) values were calculated to quantify effect sizes for the main effects and interactions and were interpreted as small (η²p < 0.06), moderate (0.06 ≤ η²p < 0.14), or large (η²p ≥ 0.14), following commonly used effect-size benchmarks (Richardson, 2011). Differences were considered significant at P < 0.05, whereas 0.05 ≤ P ≤ 0.10 was considered a statistical tendency. All analyses were performed using JAMOVI software (version 2.6.44; Sydney, Australia).

#### Relative bioavailability estimation

Prior to the estimation of relative bioavailability values (RBV), the statistical and biological validity of the dose-response assay was evaluated according to the procedures described by Finney (1978). Analysis of variance was used to test the assumptions of the assay, including equality of the intercepts between the reference and test sources at the basal response, significance of the dose-response relationship, and the presence of curvature in the response. When dose-response relationships were linear and no significant curvature was detected, RBV was estimated using the slope-ratio model. Conversely, when significant curvature was observed, indicating a nonlinear response, RBV was estimated using a nonlinear exponential regression model (Mehri et al., 2024).

#### Slope-ratio model

Relative bioavailability values of chelated-Fe, nano-Fe, and Fe oxide were estimated using a multiple slope-ratio assay with Fe sulfate (FeSO_4_) as the reference source. Several response criteria, including breast meat yield, testis weight, bone ash percentage, bone density, bone volume, tibial zinc concentration, and fecal Fe concentration, were individually evaluated using the multiple slope-ratio assay. Separate models were fitted for each response variable. Birds receiving the basal diet containing approximately 11 mg Fe/kg served as the unsupplemented control group.

The slope-ratio model was expressed as

$$Y=\;b_{0}+\;b_{1}X_{FeSO_{4}}+\;b_{1}\theta_{CH}X_{CH}+\;b_{1}\theta_{NN}X_{NN}+\;b_{1}\theta_{OX}X_{OX}$$

where Y represents the response variable under consideration, (*b_0_*) is the common intercept corresponding to the basal response, (*b_1_*) is the slope associated with FeSO_4_, and $X_{FeSO_{4}}$, $X_{CH}$, $X_{NN}$, and $X_{OX}$ represent supplemental Fe concentrations supplied from FeSO_4_, chelated-Fe, nano-Fe, and Fe oxide, respectively. Parameters $\theta_{CH}$, $\theta_{NN}$, and $\theta_{OX}$ denote the relative bioavailability coefficients of chelated-Fe, nano-Fe, and Fe oxide relative to FeSO_4_. Relative bioavailability values were expressed as percentages relative to FeSO_4_ (100 %). Model parameters were estimated by nonlinear least squares using the Levenberg–Marquardt algorithm. Approximate 95 % confidence intervals for parameter estimates were obtained from the variance-covariance matrix of the fitted model, and predicted responses were used to generate dose-response curves.

#### Nonlinear exponential model

When analysis of variance indicated significant curvature in the dose-response relationship, RBV was estimated using a nonlinear exponential regression model with FeSO_4_ as the reference source. Relative bone weight and TMY were evaluated using nonlinear exponential regression because curvature tests indicated departures from linearity.

The nonlinear model was defined as

$$Y=\;b_{0}+\;b_{1}\left[1-\text{exp}(-b_{2}\left(X_{FeSO_{4}}+\;\theta_{CH}X_{CH}+\;\theta_{NN}X_{NN}+\theta_{OX}X_{OX}\right))\right]$$

where Y represents the response variable under consideration, (*b_0_*) is the common intercept corresponding to the basal response, (*b_1_*) denotes the asymptotic maximum response, and (*b_2_*) is the exponential rate constant. Variables $X_{FeSO_{4}}$, $X_{CH}$, $X_{NN}$, and $X_{OX}$ represent supplemental Fe concentrations supplied from FeSO_4_, chelated-Fe, nano-Fe, and Fe oxide, respectively. Parameters $\theta_{CH}$, $\theta_{NN}$, and $\theta_{OX}$ represent the relative bioavailability coefficients of chelated-Fe, nano-Fe, and Fe oxide relative to FeSO_4_ and were expressed as percentages relative to the reference source. Model parameters were estimated by nonlinear least squares using the Levenberg–Marquardt algorithm. Mean observed and predicted responses were used to construct smooth dose-response curves for each Fe source. All relative bioavailability analyses were performed using R software (version 4.5.0; R Foundation for Statistical Computing, Vienna, Austria) with the packages *dplyr, ggplot2, minpack.lm, car*, and *msm*.

### Machine-learning trait sensitivity analysis

A complementary machine-learning analysis was conducted to identify biological traits with the greatest predictive information for dietary Fe concentration. The pen-level dataset comprised four Fe sources, five dietary Fe concentrations (30, 60, 90, 120, and 150 mg Fe/kg), and four complete replicate observations per Fe source × level combination. Dietary Fe concentration was the continuous response, and FeTibia, ZnTibia, FeLiver, ZnLiver, FeFecal, ZnFecal, RW, bone length, bone volume, bone density, DMb, ASHb, and ASHf were candidate predictors. Fe source and replicate identifier were not included as predictors. Analyses were performed in R version 4.5.0 using *tidyverse, tidymodels, recipes*, and *ranger*. A Random Forest regression model was fitted to accommodate nonlinear relationships and interactions. The model used 700 trees, a minimum terminal-node size of 3, and permutation-based variable importance. Predictor importance was expressed as the percentage increase in mean squared error (MSE) after randomly permuting each predictor relative to the original model. Larger positive values indicated greater predictive contribution, whereas values near or below zero indicated little or unstable incremental information. Permutation importance was interpreted as predictive contribution rather than biological causality or Fe bioavailability.

## Results

### Growth performance

Growth performance responses to dietary Fe source and supplementation level are presented in Table 3. Body weight gain was significantly affected by Fe source (*P* = 0.023) and supplementation level (*P* < 0.001), whereas the Fe source × supplementation level interaction tended to affect body weight gain (*P* = 0.078). The partial eta-squared values (η²p) indicated large effects of both Fe source (η²p = 0.155) and supplementation level (η²p = 0.382), with supplementation level accounting for a greater proportion of the explainable variation. Although the interaction did not reach statistical significance, its effect size was moderate to large (η²p = 0.256). Feed intake was not affected by Fe source (*P* = 0.185), but was significantly affected by supplementation level (*P* = 0.006; η²p = 0.269), with no significant source × level interaction (*P* = 0.331; η²p = 0.174). Feed conversion ratio was significantly affected by both Fe source (*P* = 0.012; η²p = 0.180) and supplementation level (*P* < 0.001; η²p = 0.344), and the source × level interaction was also significant (*P* = 0.014; η²p = 0.250).

**Table 3:** Effects of dietary iron source and supplementation level on growth performance of growing Japanese quail.

Fe level (mg/kg)	Source	Feed intake (g)	Gain (g)	FCR
30	FeSO_4_	338	96.5	3.51
30	Chelate	340	103.9	3.37
30	Nano	333	97.9	3.41
60	OX	335	86.9	3.87
60	FeSO_4_	325	109.7	3.00
60	Chelate	324	104.9	3.20
90	Nano	338	98.3	3.44
90	OX	326	100.0	3.32
90	FeSO_4_	325	84.4	3.85
120	Chelate	331	84.6	3.92
120	Nano	332	80.8	4.16
120	OX	323	84.6	3.83
150	FeSO_4_	314	69.4	4.54
150	Chelate	324	92.7	3.56
150	Nano	323	85.8	3.78
SEM		4.97	5.66	0.190
Probability				
Source		0.185	0.023	0.011
Level		0.006	<0.001	<0.001
Interaction		0.331	0.078	0.014
Linear		<0.001	<0.001	<0.001
Quadratic		0.910	0.510	0.370
Cubic		0.056	0.096	0.051

### Carcass traits and lymphoid organ characteristics

Carcass and relative organ characteristics are presented in Table 4. Carcass yield was significantly affected by supplementation level (*P* = 0.002), whereas the main effect of Fe source was not significant (*P* = 0.119). However, a significant Fe source × supplementation level interaction was detected (*P* = 0.016), indicating that carcass-yield responses differed according to the combination of Fe source and dietary concentration. Orthogonal polynomial contrasts revealed significant quadratic (*P* = 0.003) and cubic (*P* = 0.008) responses to increasing dietary Fe concentration. The highest carcass yield was observed in birds receiving chelated-Fe at 60 mg/kg, whereas the lowest value occurred in birds receiving nano-Fe at 30 mg/kg.

**Table 4:** Effects of dietary iron source and supplementation level on carcass characteristics and reproductive organ development of growing Japanese quail.

Fe level (mg/kg)	Source	Carcass (%)	BMY (%)	TMY (%)	Testis (%)	Bursa (%)
30	FeSO_4_	61.4	43.8	11.68	1.320	0.108
30	Chelate	60.3	44.7	11.45	0.580	0.100
30	Nano	54.0	52.7	11.22	0.345	0.078
30	OX	57.1	42.2	11.65	0.780	0.083
60	FeSO_4_	59.4	45.2	10.95	0.675	0.085
60	Chelate	69.8	46.4	11.27	0.627	0.068
60	Nano	66.3	42.1	10.83	1.018	0.075
60	OX	60.3	43.9	10.84	0.197	0.100
90	FeSO_4_	60.3	43.9	12.12	0.390	0.098
90	Chelate	64.0	45.3	12.08	1.442	0.100
90	Nano	61.5	43.7	10.98	0.850	0.090
90	OX	59.7	40.2	10.84	0.220	0.113
120	FeSO_4_	59.3	42.1	11.48	0.960	0.073
120	Chelate	62.1	43.9	11.61	1.067	0.108
120	Nano	60.2	44.2	11.39	0.895	0.108
120	OX	57.8	42.9	11.43	0.400	0.075
150	FeSO_4_	59.7	41.7	12.17	0.748	0.105
150	Chelate	55.8	43.2	11.82	1.132	0.080
150	Nano	58.5	42.4	11.02	1.893	0.100
150	OX	61.9	43.3	11.47	0.725	0.073
SEM		2.07	1.68	1.15	0.208	0.012
Probability						
Source		0.119	0.068	0.321	<0.001	0.918
Level		0.002	0.070	0.035	0.017	0.293
Interaction		0.016	0.026	0.708	<0.001	0.034
Linear		0.450	0.006	0.156	0.006	0.837
Quadratic		0.003	0.379	0.281	0.033	0.644
Cubic		0.008	0.689	0.012	0.917	0.276

Fe source (*P* = 0.068) and supplementation level (*P* = 0.070) both tended to affect BMY, whereas their interaction was significant (*P* = 0.026; η²p = 0.302). A significant linear response to increasing dietary Fe concentration was also observed (*P* = 0.006), indicating a gradual decline in BMY with increasing Fe concentration. TMY was affected by supplementation level (*P* = 0.035; η²p = 0.156), whereas neither Fe source (*P* = 0.321) nor the source × level interaction (*P* = 0.708) significantly affected this trait.

Relative liver weight was not affected by Fe source (*P* = 0.192), supplementation level (*P* = 0.128), or their interaction (*P* = 0.916), with uniformly small effect sizes. In contrast, testis relative weight was significantly affected by Fe source (*P* < 0.001), supplementation level (*P* = 0.017), and their interaction (*P* < 0.001; η²p = 0.435). Bursa weight was not affected by Fe source (*P* = 0.918) or supplementation level (*P* = 0.293), but a significant source × level interaction was detected (*P* = 0.034; η²p = 0.291). Spleen weight was not affected by Fe source (*P* = 0.457), supplementation level (*P* = 0.159), or their interaction (*P* = 0.470).

### Bone mineral composition and physical characteristics

Bone mineral composition and physical characteristics are presented in Table 5. Tibial Fe concentration was significantly affected by Fe source (*P* < 0.001), supplementation level (*P* < 0.001), and their interaction (*P* = 0.019). Chelated-Fe and nano-Fe generally resulted in greater tibial Fe concentrations than FeSO₄ and Fe oxide. Increasing dietary Fe concentration increased tibial Fe deposition, with significant linear (*P* = 0.002) and quadratic (*P* = 0.003) responses. The highest tibial Fe concentrations were observed at intermediate and high supplementation levels, particularly in birds receiving chelated-Fe, whereas Fe oxide consistently produced lower concentrations.

**Table 5:** Effects of dietary iron source and supplementation level (mg/kg) on bone mineral composition, morphometric characteristics, and fecal mineral excretion in growing Japanese quail.

Fe level	Source	Fecal-Fe	FeTibia (%)	ZnTibia (%)	FeLiver (ppm)	Length (cm)	Volume (cm^3^)	Relative weight	Density (g/cm^3^)	DMb (%)	ASHb (%)	ASHf (%)
30	FeSO_4_	373	8.60	0.333	202	5.38	0.738	0.366	1.35	67.5	36.8	11.10
30	Chelate	368	9.25	0.333	186	5.40	0.880	0.468	1.28	56.1	43.0	9.81
30	Nano	365	9.49	0.331	203	5.23	0.818	0.420	1.20	63.3	39.7	11.80
30	OX	395	7.34	0.331	181	5.10	0.768	0.357	1.19	55.1	44.4	10.34
60	FeSO_4_	378	9.35	0.335	196	5.20	0.823	0.602	1.27	72.8	38.3	13.94
60	Chelate	401	9.99	0.327	205	5.35	0.858	0.602	1.34	61.9	43.3	11.40
60	Nano	371	9.90	0.332	211	5.20	0.730	0.567	1.34	62.1	45.9	12.13
60	OX	398	8.10	0.334	200	5.08	0.668	0.533	1.23	58.4	37.4	12.37
90	FeSO_4_	421	9.32	0.333	211	5.08	0.795	0.561	1.23	72.2	40.6	13.58
90	Chelate	413	10.36	0.335	217	5.13	0.740	0.589	1.51	63.6	42.2	12.60
90	Nano	405	9.97	0.330	234	5.25	0.710	0.585	1.31	62.3	49.6	13.13
90	OX	426	8.45	0.330	201	5.25	0.795	0.579	1.34	59.8	38.4	13.92
120	FeSO_4_	435	9.35	0.332	215	5.10	0.703	0.624	1.42	70.3	41.1	12.26
120	Chelate	434	9.83	0.335	219	5.23	0.759	0.569	1.28	66.0	49.7	12.69
120	Nano	416	9.47	0.332	239	5.33	0.703	0.577	1.37	69.5	47.7	13.75
120	OX	457	8.83	0.330	215	5.15	0.640	0.560	1.27	68.0	43.9	14.08
150	FeSO_4_	446	9.46	0.339	219	5.10	0.595	0.515	1.43	71.9	44.8	14.62
150	Chelate	445	9.36	0.330	234	5.33	0.665	0.491	1.43	78.1	51.5	14.15
150	Nano	440	9.34	0.334	237	5.13	0.625	0.599	1.32	69.1	42.4	14.86
150	OX	495	9.36	0.332	208	4.95	0.650	0.567	1.26	64.3	48.3	15.58
SEM		9.54	0.282	0.002	9.05	0.123	0.0514	0.0401	0.069	3.61	3.21	0.941
Probability												
Source		<0.001	<0.001	0.200	0.001	0.121	0.109	0.647	0.089	<0.001	0.030	0.214
Level		<0.001	<0.001	0.776	<0.001	0.557	<0.001	<0.001	0.202	0.001	0.042	<0.001
Interaction		0.440	0.019	0.219	0.880	0.723	0.433	0.486	0.284	0.358	0.291	0.868
Linear		<0.001	0.002	0.296	<0.001	0.126	<0.001	<0.001	0.027	<0.001	0.003	<0.001
Quadratic		0.651	0.003	0.439	0.234	0.850	0.211	<0.001	0.500	0.817	0.597	0.520
Cubic		0.312	0.158	0.815	0.640	0.489	0.711	0.050	0.792	0.869	0.564	0.089

Tibial Zn concentration was not affected by Fe source (*P* = 0.200), supplementation level (*P* = 0.776), or their interaction (*P* = 0.219), and no significant polynomial responses were detected. ASHb percentage was affected by Fe source (*P* = 0.030) and supplementation level (*P* = 0.042), but not by their interaction (*P* = 0.291). Orthogonal polynomial analysis revealed a significant positive linear response to increasing dietary Fe concentration (*P* = 0.003). Birds receiving chelated-Fe tended to have greater ASHb percentages than those receiving FeSO₄, whereas nano-Fe produced intermediate responses.

DMb was significantly affected by Fe source (*P* < 0.001) and supplementation level (*P* = 0.001), with no significant interaction (*P* = 0.358). Birds receiving FeSO₄ generally exhibited greater DMb than those receiving chelated-, nano-, or oxide-Fe. Bone dry matter increased linearly with increasing dietary Fe concentration (*P* < 0.001). Bone length was not affected by Fe source (*P* = 0.121), supplementation level (*P* = 0.557), or their interaction (*P* = 0.723), and no significant polynomial responses were detected.

Bone density was not significantly affected by Fe source (*P* = 0.089), supplementation level (*P* = 0.202), or their interaction (*P* = 0.284); however, a significant linear response to increasing dietary Fe concentration was observed (*P* = 0.027). Bone volume was not affected by Fe source (*P* = 0.109) or the source × level interaction (*P* = 0.433), but was significantly affected by supplementation level (*P* < 0.001), with a significant negative linear response (*P* < 0.001). Relative bone weight was not affected by Fe source (*P* = 0.647) or the source × level interaction (P = 0.486), but supplementation level significantly affected this trait (*P* < 0.001; η²p = 0.492). Both linear and quadratic responses were significant (P < 0.001 for each), indicating an increase in RW with increasing Fe concentration followed by an apparent plateau at higher supplementation levels.

### Fecal ash and Fe excretion

Fecal ash and Fe concentrations are presented in Table 5. ASHf percentage was not affected by Fe source (*P* = 0.214), but increased significantly with supplementation level (*P* < 0.001), with no source × level interaction (*P* = 0.868). Orthogonal polynomial analysis demonstrated a significant positive linear response (*P* < 0.001). Fecal Fe concentration was significantly affected by Fe source (*P* < 0.001; η²p = 0.368) and supplementation level (*P* < 0.001; η²p = 0.766), whereas their interaction was not significant (*P* = 0.440). The response to increasing Fe concentration was linear (*P* < 0.001), whereas quadratic (*P* = 0.651), cubic (*P* = 0.312), and quartic (*P* = 0.338) responses were not significant. Overall, fecal Fe concentration increased from approximately 375 to 456 mg/kg dry matter as dietary Fe concentration increased from 30 to 150 mg/kg. Among Fe sources, Fe oxide produced the greatest fecal Fe concentrations, whereas nano-Fe produced the lowest values.

### Statistical validity of the relative bioavailability assays

The statistical validity of the bioavailability assays is summarized in Table 6. Significant average slopes (*P* ≤ 0.05) indicated measurable dose-related responses for gain, feed intake, TMY, testis weight, liver Fe concentration, tibial Fe concentration, bone density, RW, bone volume, ASHb percentage, and fecal Fe concentration. Significant differences among source-specific slopes were detected for feed intake, BMY, liver Fe concentration, tibial Fe concentration, ASHb percentage, and fecal Fe concentration, indicating source-dependent dose responses. Table 7

**Table 6:** Statistical validity of response criteria used for relative bioavailability estimation of dietary iron sources in growing Japanese quail.

Item	Probability (α = 0.05)																
	**Gain**	**FCR**	FI	Carcass	BMY	TMY	Testis	Bursa	**FeLiver**	ZnLiver	**FeTibia**	ZnTibia	BD	RW	Volume	Ash-b	FeFecal
Average slope	0.04	0.42	0.001	0.61	0.38	0.01	0.19	0.61	<.001	0.43	<.001	0.63	0.001	<.001	<.001	0.001	<.001
Slope difference	0.92	0.73	0.02	0.76	0.001	0.10	0.28	0.44	0.001	0.15	<.001	0.11	0.11	0.72	0.33	0.05	<.001
Blank	**0.001**	**0.01**	0.77	0.23	0.56	0.35	0.12	0.55	**0.001**	0.98	**<.001**	0.13	0.17	0.15	0.09	0.70	0.19
Intersection	0.31	0.24	0.43	0.15	0.97	0.11	0.70	0.75	0.46	0.98	**<.001**	0.93	0.82	0.08	0.23	0.38	0.65
Curvature	0.29	0.38	0.89	0.001	0.07	0.001	0.54	0.53	0.90	0.27	0.17	0.23	0.23	0.001	0.35	0.30	0.53

FCR: feed conversion ratio; FI: feed intake; BMY: breast meat yield; TMY: thigh meat yield; BD: bone density; RW: relative weight of bone; Ash-b: bone ash. Red bold values indicate response variables (e.g., gain, FCR, FeLiver, and FeTibia) that did not satisfy the statistical validity criteria for relative bioavailability estimation.

**Table 7:** Relative bioavailability (%) of chelated, nano, and oxide iron sources relative to FeSO₄ estimated by the multiple linear slope-ratio assay. Values are presented as estimate ± standard error (95 % confidence interval).

Response criterion	Chelated-Fe	Nano-Fe	Oxide Fe
Bone ash	287.8 ± 174.3 (−59.3 to 634.8)	225.8 ± 135.6 (−44.1 to 495.8)	159.6 ± 97.3 (−34.1 to 353.4)
Testis	107.7 ± 70.7 (−33.0 to 248.4)	101.8 ± 68.5 (−34.6 to 238.2)	19.4 ± 61.0 (−101.9 to 140.8)
Zn-Tibia	7.7 ± 53.0 (−97.8 to 113.1)	5.4 ± 53.5 (−101.1 to 111.8)	−32.5 ± 65.1 (−162.0 to 97.1)
Bone volume	67.7 ± 24.8 (18.3 to 117.1)	110.9 ± 29.8 (51.5 to 170.2)	113.2 ± 30.2 (53.1 to 173.3)
Bone density	116.6 ± 38.1 (40.8 to 192.5)	75.3 ± 31.4 (12.7 to 137.8)	36.1 ± 30.1 (−23.8 to 96.1)
Breast meat yield	25.1 ± 55.9 (−86.1 to 136.3)	104.0 ± 64.6 (−24.6 to 232.6)	−214.1 ± 169.2 (−550.9 to 122.7)
Fe-Fecal	101.2 ± 8.7 (83.9 to 118.4)	84.8 ± 8.0 (68.9 to 100.8)	136.3 ± 10.5 (115.4 to 157.2)

Curvature tests identified significant departures from linearity for carcass yield, TMY, testis weight, and RW (*P* < 0.05), indicating that the assumptions of a linear slope-ratio model were not fully satisfied for these traits. Consequently, nonlinear exponential models were used for RW and TMY. In contrast, bone density, bone volume, tibial Zn concentration, and fecal Fe concentration satisfied the assumptions of the multiple linear slope-ratio assay. Bone ash percentage showed acceptable but borderline statistical validity and produced highly variable RBV estimates.

### Relative bioavailability of Fe sources

For ASHb percentage, chelated-Fe produced the highest numerical RBV (287.8 %; 95 % CI: −59.3 to 634.8 %), followed by nano-Fe (225.8 %; 95 % CI: −44.1 to 495.8 %) and Fe oxide (159.6 %; 95 % CI: −34.1 to 353.4 %). The broad confidence intervals indicated substantial uncertainty, and none of the alternative Fe sources differed significantly from FeSO₄.

For bone density, chelated-Fe had an RBV of 116.6 % relative to FeSO₄ (95 % CI: 40.8–192.5 %; *P* = 0.003), whereas nano-Fe had an RBV of 75.3 % (95 % CI: 12.7–137.8 %; *P* = 0.019). Fe oxide produced the lowest numerical estimate (36.1 %; 95 % CI: −23.8 to 96.1 %; *P* = 0.234). Pairwise comparisons showed that neither chelated-Fe nor nano-Fe differed significantly from FeSO₄ (*P* > 0.05), whereas Fe oxide tended to produce a lower response.

For bone volume, chelated-Fe had an RBV of 67.7 % (95 % CI: 18.3–117.1 %; *P* = 0.008), while nano-Fe and Fe oxide produced estimates of 110.9 % (95 % CI: 51.5–170.2 %; *P* < 0.001) and 113.2 % (95 % CI: 53.1–173.3 %; *P* < 0.001), respectively. The overlapping confidence intervals indicated comparable responses between nano-Fe and Fe oxide, whereas chelated-Fe produced a lower numerical response than the reference.

For tibial Zn concentration, none of the alternative Fe sources differed significantly from FeSO₄. The RBV estimates were 7.7 % for chelated-Fe (95 % CI: −97.8 to 113.1 %), 5.4 % for nano-Fe (95 % CI: −101.1 to 111.8 %), and −32.5 % for Fe oxide (95 % CI: −162.0 to 97.1 %). The wide confidence intervals indicated substantial uncertainty in these estimates.

Because fecal Fe concentration is an inverse indicator of apparent Fe utilization, greater fecal Fe excretion corresponds to lower Fe absorption and retention. For this endpoint, higher RBV values therefore indicate poorer Fe utilization. Fe oxide produced the greatest relative fecal Fe excretion, with an RBV of 136.3 % (95 % CI: 115.4–157.2 %; *P* < 0.001), indicating the poorest apparent Fe utilization. Chelated-Fe had a response comparable to FeSO₄ (101.2 %; 95 % CI: 83.9–118.4 %), whereas nano-Fe had the lowest relative fecal Fe excretion (84.8 %; 95 % CI: 68.9–100.8 %; *P* < 0.001). Pairwise comparisons showed no difference between chelated-Fe and FeSO₄ (*P* = 0.894), whereas nano-Fe tended to reduce fecal Fe excretion (*P* = 0.062). Fe oxide significantly increased fecal Fe losses relative to FeSO₄ (*P* < 0.001).

For BMY, chelated-Fe yielded an RBV of 25.1 % (95 % CI: −86.1 to 136.3 %), nano-Fe 104.0 % (95 % CI: −24.6 to 232.6 %), and Fe oxide −214.1 % (95 % CI: −550.9 to 122.7 %). Because the average slope was not significant and source-specific slopes differed (Table 6), the assumptions of the linear slope-ratio model were only partially satisfied. These estimates therefore provide insufficient evidence for meaningful differences among Fe sources for breast muscle development.

For testis weight, chelated-Fe and nano-Fe produced similar numerical RBV estimates of 107.7 % (95 % CI: −33.0 to 248.4 %) and 101.8 % (95 % CI: −34.6 to 238.2 %), respectively, whereas Fe oxide yielded 19.4 % (95 % CI: −101.9 to 140.8 %). The confidence intervals were broad and included the reference value. In addition, significant curvature was detected, indicating that the assumptions of the linear slope-ratio model were not fully satisfied. These estimates should therefore be considered exploratory.

### Relative bioavailability based on the nonlinear exponential model

Relative bioavailability estimates from the nonlinear exponential model are presented in Table 8. Because curvature tests indicated nonlinear responses for relative bone weight and TMY, these traits were analyzed using nonlinear regression rather than the linear slope-ratio model.

**Table 8:** Relative bioavailability (%) of chelated, nano, and oxide iron sources relative to FeSO₄ estimated using nonlinear exponential regression models. Values are presented as estimates ± standard errors with approximate 95 % confidence intervals.

Response criterion	Chelated-Fe	Nano-Fe	Oxide Fe
Relative weight of bone	151.2 ± 64.7 (22.5 to 280)	113.2 ± 43.8 (25.9 to 200.4)	79.8 ± 28.5 (23.1 to 136.4)
Thigh meat yield	118.0 ± 90.5 (−62.2 to 298.2)	59.3 ± 36.5 (−13.4 to 132)	44.2 ± 26.2 (−7.9 to 96.3)

For RW, chelated-Fe produced the highest numerical RBV (151.2 %; 95 % CI: 22.5–280.0 %), followed by nano-Fe (113.2 %; 95 % CI: 25.9–200.4 %) and Fe oxide (79.8 %; 95 % CI: 23.1–136.4 %). The fitted curves showed a rapid increase in RW at lower supplementation levels followed by a plateau at higher Fe concentrations. However, the confidence intervals were broad and overlapping, indicating uncertainty in the relative ranking of Fe sources.

For TMY, chelated-Fe produced the highest numerical RBV (118.0 %; 95 % CI: −62.2 to 298.2 %), followed by nano-Fe (59.3 %; 95 % CI: −13.4 to 132.0 %) and Fe oxide (44.2 %; 95 % CI: −7.9 to 96.3 %). The fitted curves showed an initial increase followed by a plateau at higher supplementation levels. Nevertheless, the broad and overlapping confidence intervals indicated substantial uncertainty and did not support definitive conclusions regarding differences in biological effectiveness among Fe sources. Fig. 1, Fig. 2

**Fig. 1:**
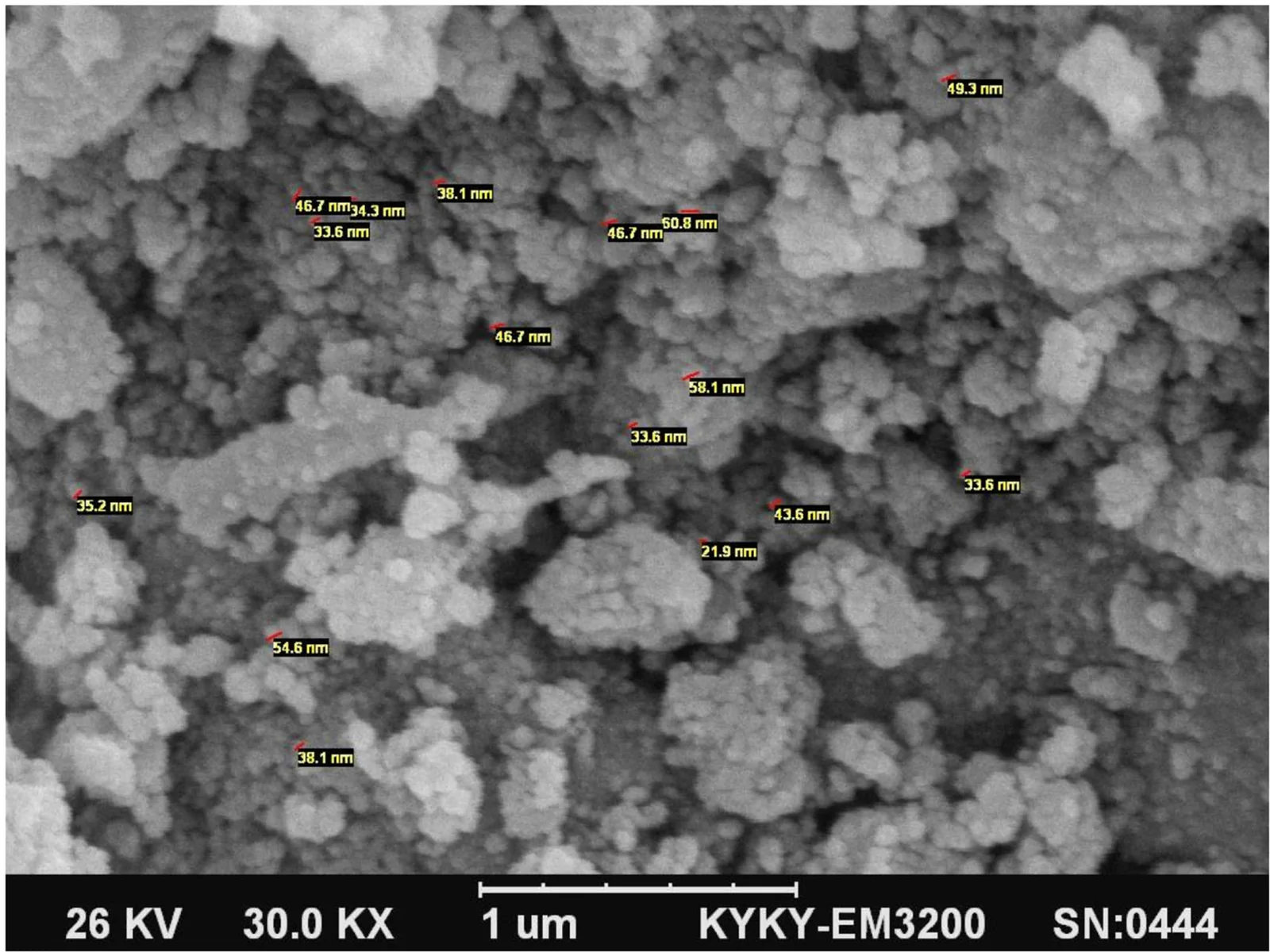
Scanning electron microscopy (SEM) of iron-nanoparticles with particle size of 20-30 nm. Left to right, the specifications SEM image are as fllows: 1. The applied voltage, used to generate a stronger magnetic field to accelerate electrons and capture the photo. 2. The magnification factor of the image, indicating the degree of enlargement. In this photo, the image was taken with a magnification of 30,000 times. 3. The scale of the image, which can be used to estimate the size of the image's details. 4. The name and model of the SEM device used. 5. The image computer number or snapshot ID.

**Fig. 2:**
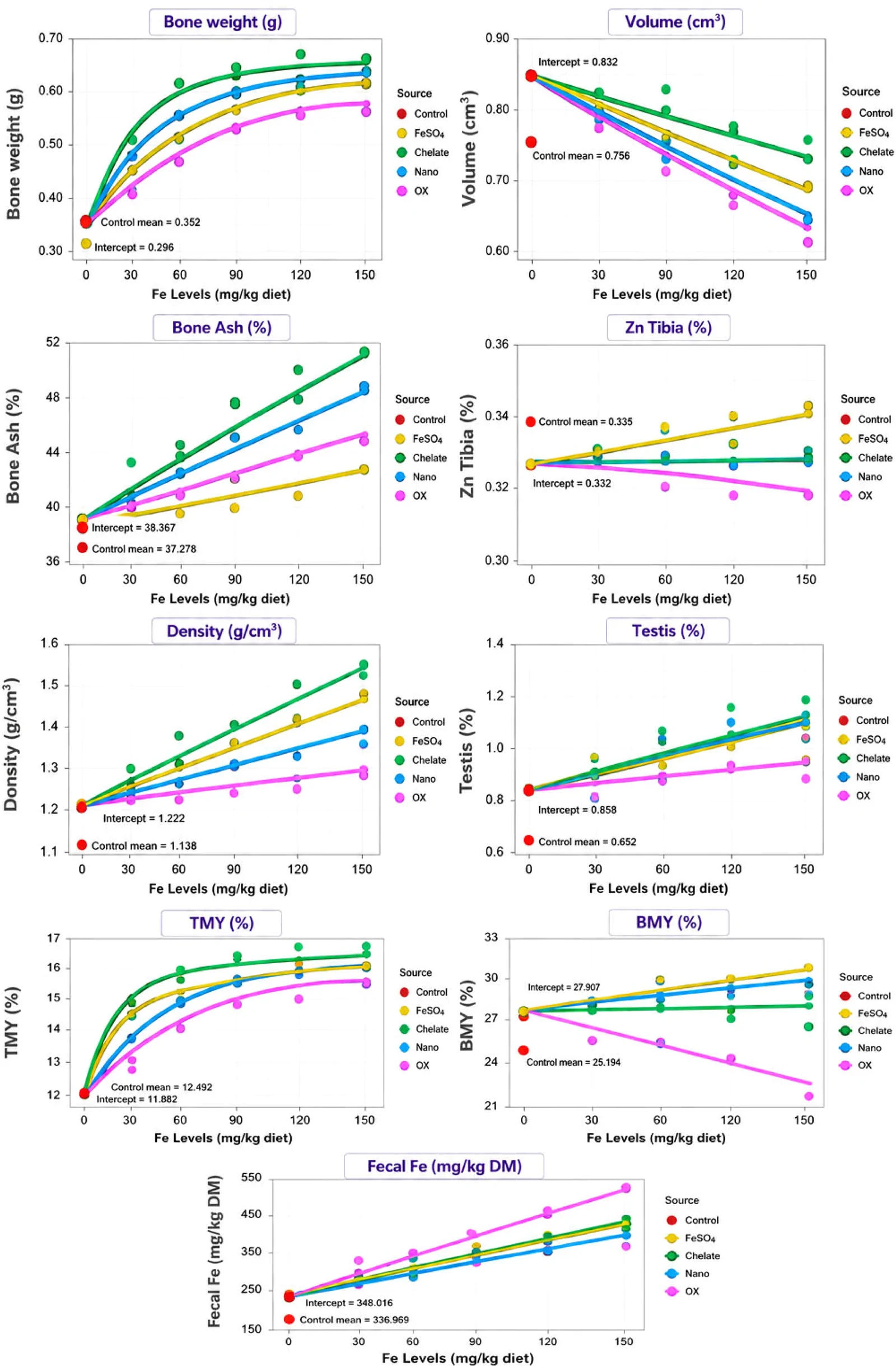
Fitted relative bioavailability (RBV) curves describing the response of bone, carcass, reproductive, and mineralization traits to increasing dietary iron supplementation from FeSO₄, chelated, nano, and oxide sources in male Japanese quail. Data points represent treatment means, and solid lines represent the best-fitting slope-ratio or exponential RBV models used to estimate source-specific bioavailability relative to FeSO₄ (RBV = 100 %). Responses include bone weight, bone volume, bone ash, tibial zinc concentration, bone density, testis weight, thigh meat yield, breast meat yield, and fecal iron concentration. Control means and common intercepts are shown for each trait to facilitate comparison among iron sources.

### Machine-learning trait sensitivity to dietary Fe

The Random Forest permutation analysis revealed marked differences in the predictive information contained in the measured biological traits (Fig. 3). FeFecal was the dominant predictor of dietary Fe concentration, with permutation producing an approximately 75 % increase in MSE. RW was the second most informative trait (approximately 54 %), followed by ASHf (44 %) and FeLiver (38 %). Intermediate importance was observed for VOL (33 %), ZnFecal (30 %), DMb (29 %), and FeTibia (28 %), whereas ASHb contributed less strongly (17 %). DEN showed only a small contribution (approximately 4 %), and ZnLiver provided essentially no incremental predictive information. ZnTibia and length showed negative permutation importance (approximately −4 % and −10 %, respectively), indicating little or unstable incremental predictive information after the other traits were considered. The prominence of FeFecal was consistent with the conventional analysis, in which fecal Fe concentration increased progressively with dietary Fe concentration and was significantly influenced by both Fe source and supplementation level. The conventional analysis also demonstrated source-dependent differences in fecal Fe excretion, with Fe oxide producing the greatest fecal Fe concentrations and nano-Fe the lowest. The four most informative traits, FeFecal, RW, ASHf, and FeLiver, also displayed distinct response trajectories across the dietary Fe gradient (Fig. 3), demonstrating that the machine-learning ranking reflected coordinated changes in fecal Fe excretion, bone phenotype, and hepatic Fe storage rather than variation in a single endpoint.

**Fig. 3:**
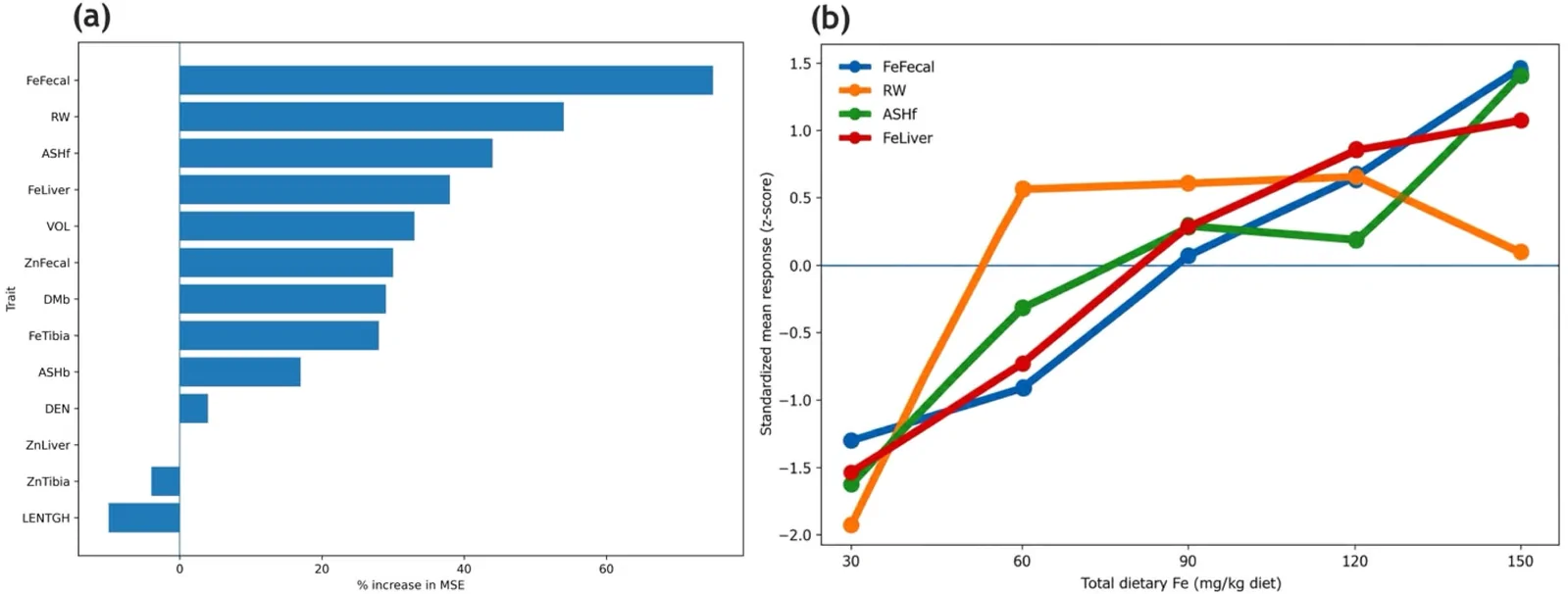
Random Forest permutation importance and response trajectories of biological traits across dietary Fe levels. (a) Permutation importance of individual traits for prediction of dietary Fe concentration, expressed as percentage increase in mean squared error (MSE) following permutation. (b) Observed response trajectories of the four highest-ranked traits (FeFecal, RW, ASHf, and FeLiver), presented as standardized treatment means (z-scores) across dietary Fe concentrations.

## Discussion

The present study demonstrated that dietary Fe concentration was the major determinant of quail responses, whereas the chemical form of Fe exerted more selective effects that became particularly evident in tissue mineral deposition, bone characteristics, and fecal Fe excretion. Significant interactions between source and level of dietary Fe and nonlinear responses were observed for several variables, indicating that the biological value of each Fe source depended on the level of supplementation rather than remaining constant across the range examined. These findings align with the idea that mineral bioavailability varies by endpoint and that Fe absorption/deposition involves regulated, saturable mechanisms.

### Growth performance and feed efficiency

Dietary Fe concentration accounted for a larger proportion of variation in gain, FI, and FCR than Fe source, indicating that satisfying the physiological requirement for Fe is more important than the specific chemical form for maintaining productive performance. Fe participates in oxygen transport, mitochondrial respiration, electron transfer reactions, DNA synthesis, and numerous enzyme systems, making adequate Fe status essential for efficient metabolism and growth (Sharma et al., 2023; Yang et al., 2024). Once minimum requirements are met, however, birds are capable of maintaining productive performance over a relatively broad range of Fe intakes through homeostatic regulation, making growth traits relatively insensitive indicators of differences in bioavailability (Byrne et al., 2022; Cao et al., 1996; Richards et al., 2010). These findings agree with those of Cao et al. (1996), who demonstrated that tissue Fe concentration was more sensitive than growth performance for distinguishing among supplemental Fe sources. Similarly, Ma et al. (2014) reported that Fe proteinate possessed greater relative bioavailability than FeSO_4_ when assessed using hemoglobin Fe and total body Fe, whereas growth responses alone provided limited discrimination among sources. Therefore, performance traits should be interpreted primarily as indicators of nutritional adequacy rather than definitive measures of bioavailability. Another explanation for the relatively modest effects of Fe source on growth performance is the existence of robust physiological mechanisms regulating whole-body Fe homeostasis. Intestinal Fe absorption is tightly controlled by systemic Fe status through the hepcidin–ferroportin axis. When body Fe stores are adequate, hepatic hepcidin production increases, reducing ferroportin activity in enterocytes and thereby limiting further Fe absorption regardless of dietary supply (Byrne et al., 2022; Ganz, 2013). Consequently, additional improvements in intestinal Fe availability are not necessarily translated into proportional improvements in growth. This regulatory system explains why birds receiving chelated-Fe or nano-Fe exhibited improved tissue Fe deposition without corresponding increases in body weight gain across all supplementation levels. Similar observations have been reported in broilers, where tissue Fe concentration and hematological variables responded more sensitively than productive performance to increasing Fe bioavailability (Cao et al., 1996; Ma et al., 2014). Therefore, growth performance should be regarded as a relatively insensitive endpoint for discriminating among Fe sources under conditions where basal dietary requirements are largely satisfied.

Feed intake was affected by dietary Fe concentration but not by Fe source, suggesting that Fe exerts its principal effects through postabsorptive metabolic processes rather than appetite regulation. This observation is biologically reasonable because Fe deficiency initially impairs oxygen delivery and oxidative metabolism rather than feed consumption (Udipi et al., 2012). Consequently, improvements in growth performance most likely resulted from enhanced metabolic efficiency rather than increased voluntary feed intake. The significant *source × level* interaction observed for FCR indicates differences in utilization efficiency among Fe forms. Chelated-Fe generally supported better feed efficiency than Fe oxide, whereas nano-Fe produced intermediate or favorable responses. The superiority of chelated-Fe probably reflects differences in physicochemical properties and gastrointestinal stability. In the acidic conditions of the upper gastrointestinal tract, inorganic mineral salts readily dissociate, resulting ions then become susceptible to interactions with phytate, phosphates, carbonates, and hydroxides, leading to reduced solubility and impaired absorption (Byrne et al., 2022). In contrast, organic complexes maintain greater stability over a wider pH range and are protected against antagonistic interactions, thereby improving mineral availability. Similar improvements in tissue retention and productive performance have been reported for organically complexed trace minerals in broilers (Bao et al., 2007; Jarosz et al., 2021). It should also be recognized that previous studies have not consistently demonstrated superior growth responses to organic or nano-sized Fe sources. Although improvements in tissue Fe retention are frequently observed, productive performance often remains unchanged when birds are not Fe deficient (Bai et al., 2021; Jarosz et al., 2021). Such inconsistencies probably reflect differences in dietary Fe concentration, bird age, genetic background, dietary antagonists, and experimental duration. Consequently, discrepancies among studies should not necessarily be interpreted as conflicting evidence regarding Fe bioavailability but rather as differences in the physiological sensitivity of the response variables evaluated. This highlights the importance of selecting appropriate biological endpoints when comparing Fe sources.

### Carcass characteristics and organ development

Carcass yield responded to dietary Fe concentration and exhibited *source × level* interactions, indicating that the optimal level of supplementation differed among Fe sources. The highest carcass yield occurred with chelated-Fe at intermediate concentrations, suggesting that excessive supplementation does not necessarily confer additional benefits. Nonlinear responses of essential nutrients are expected because biological responses increase rapidly under deficient conditions and approach a plateau as requirements become satisfied (Tako et al., 2010). Previous studies have likewise reported selective effects of Fe supplementation on carcass characteristics. Basharat et al. (2023) demonstrated improvements in feed efficiency and intestinal morphology with Fe nanoparticles but observed limited changes in carcass traits. Similarly, Almeldin et al. (2024) found that green Fe nanoparticles improved growth performance and physiological status without producing uniform responses across all carcass characteristics. Therefore, Fe appears to influence carcass composition indirectly through improved nutrient utilization and metabolic efficiency rather than through direct effects on muscle accretion.

Breast and thigh yields responded differently to dietary Fe, suggesting tissue-specific sensitivity. Breast muscle is composed predominantly of glycolytic fibers, whereas thigh muscle contains more oxidative fibers that rely heavily on oxygen supply and mitochondrial activity (Lilburn et al., 2019; Weng et al., 2022). Since Fe is intimately involved in oxygen transport and cellular respiration, differential responses among muscle groups are biologically plausible. Similar tissue-specific effects have been described previously for trace mineral supplementation (Richards et al., 2010). The relatively small effects of Fe supplementation on carcass characteristics further support the concept that carcass composition is influenced by multiple nutritional and endocrine factors beyond Fe nutrition alone. Muscle accretion primarily depends upon adequate amino acid supply, energy intake, endocrine regulation, and satellite cell activity, whereas Fe mainly facilitates oxygen transport and mitochondrial ATP production required to sustain these anabolic processes (Lilburn et al., 2019). Therefore, improvements in Fe utilization may enhance metabolic efficiency without necessarily altering carcass yield. Similar observations have been reported for other trace minerals, including Zn and Cu, where tissue mineral concentrations changed substantially despite limited effects on carcass composition (Richards et al., 2010). Such findings reinforce that carcass traits possess lower sensitivity for assessing trace mineral bioavailability than tissue mineral deposition.

Among internal organs, testes weight exhibited one of the strongest responses to dietary treatments. Fe is required for mitochondrial energy production, DNA synthesis, and steroidogenesis; therefore, reproductive tissues are expected to be highly sensitive to alterations in Fe status. Both Fe deficiency and excess may induce oxidative stress and impair reproductive development (Harrer et al., 2025). Conversely, liver, spleen, and bursa weights remained relatively stable. This lack of response should not be interpreted as evidence of equivalent biological effects because gross organ weights are relatively insensitive indicators influenced by numerous physiological factors. Previous bioavailability studies have similarly demonstrated that tissue mineral concentrations and biochemical indices provide greater sensitivity than organ weights (Cao et al., 1996; Ma et al., 2014).

### Bone characteristics and tissue Fe deposition

Bone-related measurements provided some of the clearest evidence of differences among Fe sources and appeared to be more sensitive indicators of Fe bioavailability than growth performance. Tibial Fe concentration, bone ash, and bone density responded significantly to Fe source and supplementation level, with chelated-Fe and nano-Fe generally producing greater responses than FeSO_4_ and especially Fe oxide. These findings agree with previous studies showing that tissue mineral concentrations represent more reliable criteria for assessing Fe bioavailability than body weight gain or feed efficiency (Byrne et al., 2022; Cao et al., 1996; Ma et al., 2014). The response criteria strongly influence relative bioavailability estimates, and tissue deposition variables generally provide superior discrimination among mineral sources compared with performance traits (Byrne et al., 2022). The observed improvements in bone characteristics may also reflect the essential role of Fe in extracellular matrix formation. Iron serves as a cofactor for prolyl and lysyl hydroxylases, enzymes responsible for collagen hydroxylation during collagen maturation. Proper collagen cross-linking is necessary for the formation of a mechanically competent bone matrix capable of supporting subsequent mineral deposition (Bhowmik et al., 2023). Consequently, improved Fe availability may influence bone quality through effects on organic matrix synthesis before measurable changes occur in bone mineral concentration. This mechanism may partly explain why bone density responded more consistently than bone ash percentage in the present study. Bone density integrates changes in both mineral deposition and bone architecture and therefore may represent a more biologically relevant endpoint for evaluating Fe bioavailability than ash content alone. Tibial Zn concentration was evaluated as a complementary indicator of potential Fe–Zn interactions rather than as a direct measure of Fe bioavailability. Although Fe and Zn can interact during intestinal absorption and systemic homeostasis, their absorption pathways are not identical. Therefore, the absence of significant effects of Fe source or supplementation level on ZnTibia in the present study suggests that the Fe treatments did not substantially alter Zn deposition in bone under the conditions tested.

The superior responses observed with chelated-Fe probably reflect greater physicochemical stability and improved resistance to antagonistic interactions within the gastrointestinal tract. Organic mineral complexes are less susceptible to precipitation with phytate, phosphates, carbonates, and hydroxides, thereby maintaining greater solubility over a wide pH range (Byrne et al., 2022; Richards et al., 2010). Moreover, organically bound minerals may utilize amino acid or peptide transport pathways, further enhancing absorption efficiency. In contrast, inorganic oxides possess low solubility and limited dissociation characteristics, restricting the availability of absorbable Fe²⁺ ions and ultimately reducing tissue deposition (Bai et al., 2021; Byrne et al., 2022).

Bone ash percentage increased with Fe supplementation level, with chelated-Fe generally producing the highest values. Although Fe is not a major structural constituent of bone, it is involved in collagen synthesis, osteoblast activity, and numerous metalloenzymes participating in bone formation (Bhowmik et al., 2023; Yang et al., 2011). Improved Fe availability may therefore indirectly enhance bone mineralization and facilitate the utilization of calcium and phosphorus. Similar improvements in bone quality and mineral retention have been reported with organic trace minerals in poultry (Bao et al., 2007; Nys et al., 2018). These findings are particularly important because bone abnormalities remain among the most important welfare and economic issues in modern broiler production.

Bone density increased with dietary Fe concentration, whereas bone volume declined and relative bone weight exhibited nonlinear responses. These observations suggest the formation of denser and more compact bone rather than larger bone structures. Such nonlinear responses are consistent with saturation kinetics of nutrient deposition and indicate that rapid improvements occur at lower levels of supplementation, followed by diminishing responses once requirements have been satisfied. Thus, bone density and tibial Fe concentration appear to be among the most reliable indicators for evaluating Fe bioavailability.

### Fecal Fe excretion and environmental implications

Fecal Fe concentration was one of the most discriminating response variables in the present study. Fe concentration in excreta increased linearly with dietary Fe supplementation, with Fe oxide producing the greatest fecal Fe concentrations and nano-Fe producing the lowest. Since unabsorbed Fe is eliminated primarily through the feces, fecal Fe concentration provides an inverse indicator of apparent Fe utilization. The lower fecal Fe concentrations observed with nano-Fe are consistent with greater apparent Fe retention and utilization, whereas the higher excretion associated with Fe oxide is consistent with lower apparent Fe utilization. The poor performance of Fe oxide is consistent with the lower bioavailability generally associated with oxide forms compared with sulfate and organic forms. Byrne et al. (2022) emphasized that oxide sources possess lower solubility and release smaller amounts of absorbable ions, thereby reducing their efficiency. Sulfates are generally more bioavailable than oxides, whereas organic trace minerals often exhibit even greater biological availability because of improved chemical stability and protection from antagonistic interactions (Byrne et al., 2022; Richards et al., 2010). Nano-sized minerals possess unique physicochemical characteristics that distinguish them from conventional mineral sources. Their extremely large surface area, enhanced dissolution characteristics, and prolonged retention time within the gastrointestinal tract may improve absorption and tissue utilization (Hassan et al., 2020). Furthermore, the reduced particle size minimizes antagonistic interactions and may facilitate cellular uptake, thereby decreasing mineral losses through excretion. Consistent with the present results, previous studies have shown that nano-Fe supplementation can improve Fe utilization and selected productive traits. Beneficial effects on growth performance, tissue mineral status, and intestinal morphology have been reported following supplementation with Fe nanoparticles (Almeldin et al., 2024; Basharat et al., 2023; Saki et al., 2014). The reduction in fecal mineral excretion observed with nano-Fe and chelated-Fe has important environmental implications. Excessive trace mineral excretion contributes to the accumulation of metals in poultry litter and agricultural soils, increasing the risk of environmental contamination. Because conventional inorganic minerals are often poorly retained, they are frequently supplemented with large safety margins, resulting in unnecessary mineral losses. Improved bioavailability not only enhances nutrient utilization but also contributes to more sustainable poultry production systems (Bao et al., 2007; Nys et al., 2018). Beyond its nutritional implications, reduced fecal Fe excretion may contribute to improved environmental sustainability in poultry production. Commercial poultry diets often contain substantial safety margins for trace mineral supplementation because of uncertainty regarding mineral bioavailability. However, poorly absorbed minerals are largely excreted and subsequently accumulate in poultry litter, increasing the loading of trace elements into agricultural soils following land application (Bao et al., 2007; Nys et al., 2018). Although Fe generally poses lower environmental concern than Cu or Zn, excessive mineral accumulation may alter soil microbial activity and nutrient cycling over prolonged periods. Therefore, improvements in Fe utilization achieved through chelated or nano-sized sources may permit lower dietary supplementation rates while maintaining adequate physiological responses, thereby improving nutrient efficiency and supporting more sustainable poultry production systems.

### Relative bioavailability and endpoint dependency

Relative bioavailability estimates obtained in the present study varied substantially among response criteria and did not support a consistent ranking of Fe sources across all biological endpoints. The favorable responses observed for chelated-Fe in some endpoints are broadly consistent with previous reports of enhanced bioavailability of organic Fe sources. Ma et al. (2014) reported that Fe proteinate exhibited greater relative bioavailability than FeSO_4_ based on hemoglobin Fe and total body Fe measurements, whereas Jarosz et al. (2021) observed higher serum and liver Fe concentrations in broilers receiving Fe glycine chelates. However, relative bioavailability values varied substantially among response criteria, emphasizing that bioavailability should not be regarded as a fixed characteristic. The relative bioavailability estimates are influenced by species, age, physiological status, dietary composition, antagonistic interactions, response criteria, and statistical methodology (Byrne et al., 2022; Mehri et al., 2024). One of the most important findings of the present study is that the estimated RBV varied considerably according to the biological response criterion selected. This observation supports the concept that RBV is not an intrinsic characteristic of a mineral source but rather an estimate reflecting the interaction between physiological regulation, experimental conditions, and statistical methodology (Finney, 1978; Mehri et al., 2024). Growth-related variables integrate numerous biological processes unrelated to Fe metabolism and therefore exhibit relatively low sensitivity to differences in mineral absorption. Conversely, tissue mineral deposition and fecal mineral excretion are more directly associated with Fe absorption and retention, allowing greater discrimination among Fe sources. Consequently, meaningful comparisons of RBV values among published studies require careful consideration of both the response variable and the statistical model employed. Failure to recognize these methodological differences may partly explain the substantial variability reported in the literature for identical mineral sources. Consequently, direct comparison of RBV values among studies should be approached with caution. The substantial variation observed among traits in the present study supports this concept and demonstrates that response-dependent interpretation is essential. Bone density, tibial Fe concentration, bone volume, and fecal Fe concentration provided the most consistent estimates of relative bioavailability, whereas breast yield, testes weight, and bone ash produced wider confidence intervals and less stable estimates. These findings support the recommendation of Byrne et al. (2022) that tissue mineral deposition and excretion variables should be prioritized over growth performance traits when evaluating trace mineral bioavailability. Furthermore, several variables exhibited nonlinear responses, indicating that assumptions required for linear slope-ratio assays were not always satisfied. Since nutrient absorption and tissue deposition are regulated by homeostatic mechanisms, dose-response relationships frequently deviate from linearity. Therefore, the use of nonlinear models where appropriate represents an important strength of the present study and avoids misinterpretation of relative bioavailability estimates (Mehri et al., 2024).

The precision of the estimated RBVs varied considerably among response criteria and should be considered when interpreting differences among Fe sources. Several estimates were associated with wide 95 % confidence intervals, including values that crossed zero or spanned more than 200 percentage points. For example, the estimated RBV of chelated-Fe based on bone ash was 287.8 %, but the 95 % CI ranged from −59.3 to 634.8 %, whereas the nonlinear estimate for relative bone weight was 151.2 % (95 % CI: 22.5–280.0 %). Such wide intervals indicate substantial uncertainty in the magnitude of these estimates and limit the strength of source rankings based solely on point estimates. Therefore, numerical RBV values greater than 100 % should not necessarily be interpreted as evidence of superior biological availability relative to FeSO₄. Rather, the results indicate endpoint-specific differences in biological response, with the strength of evidence depending on both the estimated effect and its precision.

### Mechanisms underlying source-dependent differences in Fe bioavailability

The source-dependent responses observed in the present study can be explained by the complex and tightly regulated mechanisms governing Fe absorption and homeostasis. Fe uptake occurs primarily in the proximal small intestine and involves several transporters and regulatory proteins that prevent both deficiency and excessive accumulation (Byrne et al., 2022; Tako et al., 2010). Ferric Fe (Fe^3+^) is poorly absorbed and must first be reduced to the ferrous form (Fe^2+^) by ferrireductases such as duodenal cytochrome b (Dcytb). The resulting Fe^2+^ is transported into enterocytes through divalent metal transporter 1 (DMT1), which serves as the principal pathway for intestinal Fe uptake. Once inside the enterocyte, Fe may either be stored in ferritin or exported across the basolateral membrane by ferroportin for incorporation into transferrin and subsequent distribution to peripheral tissues (Ohgami et al., 2005).

Fe absorption is tightly controlled by hepcidin, a peptide hormone synthesized in the liver that acts as the central regulator of systemic Fe homeostasis (Yang et al., 2011). Under conditions of adequate Fe status, increased hepcidin production suppresses ferroportin activity, thereby reducing Fe export from enterocytes and limiting further absorption (Przybyszewska et al., 2014). Excess intracellular Fe is temporarily stored as ferritin, thereby limiting oxidative damage while maintaining Fe availability for future metabolic demands (Yang et al., 2011). Consequently, Fe absorption becomes progressively saturated as dietary Fe supply increases, explaining why several variables in the present study exhibited nonlinear responses and why improvements in tissue Fe deposition were not always accompanied by proportional increases in growth performance (Tako et al., 2010).

Differences in source chemistry provide a biological explanation for the variation observed among Fe sources. Sulfate sources readily dissociate in the acidic environment of the proventriculus and gizzard, releasing ionic Fe that subsequently becomes susceptible to precipitation with phytate, phosphates, carbonates, and hydroxides as intestinal pH increases. These interactions reduce Fe solubility and decrease the amount of Fe available for absorption (Byrne et al., 2022; Richards et al., 2010). Oxide forms are even less soluble and consequently exhibit lower biological availability. This explanation is consistent with the poor tissue deposition and greater fecal Fe excretion observed with Fe oxide in the present study. Similar differences between oxide and sulfate forms have been reported previously, and sulfates are generally recognized as possessing greater bioavailability than oxides as extensively reviewed by Byrne et al. (2022). In contrast, chelated-Fe possesses greater physicochemical stability and is protected from undesirable interactions within the gastrointestinal tract. Organic ligands maintain Fe in soluble forms over a broad pH range and may facilitate absorption through amino acid or peptide transport systems in addition to conventional mineral transport pathways. Differences in ligand characteristics, chelation strength, molecular size, and pH stability may further influence dissociation rates and absorption efficiency (Byrne et al., 2022; Richards et al., 2010). These characteristics may partly explain the favorable responses observed for tibial Fe concentration, bone density, and carcass yield with chelated-Fe, although endpoint-specific RBV estimates and their confidence intervals indicate that these responses should not be interpreted as evidence of consistent superiority over FeSO_4_. Similar improvements have been reported for Fe proteinate and Fe glycine chelates in broilers (Jarosz et al., 2021; Ma et al., 2014). The response to nano-Fe was particularly interesting because nano-Fe frequently improved tissue retention and reduced fecal Fe losses without consistently producing superior growth or carcass characteristics. Nanoparticles possess unique physical and chemical properties, including increased surface area, enhanced dissolution kinetics, prolonged retention time within the intestine, and reduced susceptibility to antagonistic interactions (Hassan et al., 2020). Because particles smaller than several hundred nanometers may interact differently with biological membranes, nano-sized minerals have been proposed to exhibit improved absorption and tissue distribution compared with conventional mineral forms. Nevertheless, enhanced absorption efficiency does not necessarily translate into improved performance, because productive responses are ultimately constrained by physiological requirements and homeostatic regulation. This concept explains why nano-Fe improved mineral retention without uniformly outperforming chelated-Fe or FeSO_4_ across all measured traits (Almeldin et al., 2024; Basharat et al., 2023). Despite the promising responses observed with nano-Fe, potential safety aspects should also be considered. Nanoparticles possess physicochemical properties that differ substantially from conventional mineral sources and may exhibit altered tissue distribution, cellular uptake, and biological persistence. Although numerous poultry studies have reported beneficial effects of nano-Fe on growth performance and mineral utilization, excessive accumulation could theoretically induce oxidative stress through increased production of reactive oxygen species if antioxidant defenses become overwhelmed (Hassan et al., 2020). The present study did not evaluate oxidative stress biomarkers or tissue pathology, and therefore no conclusions can be drawn regarding long-term safety. Future investigations integrating molecular, histopathological, and toxicological endpoints will be valuable for defining optimal supplementation strategies that maximize Fe utilization while maintaining biological safety.

### Machine-learning analysis of trait responses to dietary Fe

Fig. 3a shows the Random Forest permutation importance of the measured traits for predicting dietary Fe concentration, whereas Fig. 3b illustrates the observed dose-response patterns of the four highest-ranked traits: FeFecal, RW, ASHf, and FeLiver. Because the responses in Fig. 3b are presented as standardized means (z-scores), the figure reflects relative changes across dietary Fe concentrations rather than differences in measurement units. FeFecal showed the clearest monotonic increase, rising progressively from the lowest to the highest Fe concentration. This response is consistent with greater fecal Fe excretion as dietary Fe supply increases and agrees with its high predictive importance in Fig. 3a. FeLiver also generally increased with dietary Fe, although the response was less uniform, with a smaller increment at the highest concentration. Tissue Fe accumulation has been reported to respond positively to increasing dietary Fe, supporting the use of tissue Fe as an indicator of Fe exposure or utilization (Van Ravenswaay et al., 2001; Zhang et al., 2016).However, the present FeLiver pattern should be interpreted as a characteristic of this experiment rather than as a direct replication of those studies. In contrast, RW and ASHf showed nonlinear trajectories in Fig. 3b. RW increased at low-to-intermediate Fe concentrations but declined at the highest level, whereas ASHf increased initially, changed little at the intermediate concentration, and then increased markedly at 150 mg/kg. These patterns demonstrate that predictive importance does not necessarily require a monotonic dose-response relationship. Their contribution to the Random Forest model may therefore reflect their ability to distinguish different portions of the Fe-dose range and/or their association with the broader physiological response to Fe supplementation.

### Strengths and Limitations

Multiple Fe sources were evaluated across a broad range of supplementation levels, allowing characterization of *source × level* interactions and nonlinear dose-response relationships. Furthermore, the study incorporated a wide spectrum of response criteria encompassing growth performance, carcass characteristics, reproductive organs, bone variables, tissue mineral deposition, and fecal Fe excretion. Such comprehensive evaluation provides a more complete assessment of bioavailability than studies relying exclusively on growth responses. Another important strength was the application of statistical validity tests and nonlinear regression models when assumptions required for conventional slope-ratio analysis were violated (Mehri et al., 2024). Nevertheless, several limitations should also be acknowledged. Some response criteria produced relatively wide confidence intervals and imprecise relative bioavailability estimates, particularly those exhibiting nonlinear responses. In addition, direct measurements of Fe metabolism, including hepcidin concentration and expression of DMT1 and ferroportin, were not determined. Such measurements would provide valuable mechanistic insight into the physiological basis of source-dependent responses. Furthermore, the present results are specific to the genotype, age, and dietary conditions employed and should not necessarily be extrapolated directly to all commercial production systems. Because relative bioavailability is influenced by species, age, dietary composition, antagonistic interactions, and response criteria, caution is warranted when comparing values among studies (Byrne et al., 2022). Additional mechanistic measurements would further strengthen interpretation of the present findings. Determination of hematological indicators such as hemoglobin concentration, serum ferritin, transferrin saturation, and total Fe-binding capacity would provide complementary evidence regarding systemic Fe status. Likewise, quantification of intestinal DMT1, ferroportin, and hepcidin expression could clarify the molecular mechanisms underlying the observed source-dependent differences in Fe utilization. Measurement of oxidative stress biomarkers, including superoxide dismutase, glutathione peroxidase, and malondialdehyde, would also help distinguish beneficial improvements in Fe availability from potential pro-oxidant effects associated with excessive Fe accumulation. Integration of these physiological and molecular measurements with conventional bioavailability assays would provide a more comprehensive understanding of Fe metabolism in poultry.

Another limitation of the present study is that the lowest experimental Fe concentration (30 mg/kg diet) was below the 120 mg/kg Fe requirement reported by NRC (1994) for growing Japanese quail. Importantly, the experimental concentrations of 30, 60, 90, 120, and 150 mg Fe/kg represented the intended final dietary Fe concentrations rather than supplemental amounts added to the approximately 11 mg Fe/kg present in the basal diet. Nevertheless, the relatively low Fe concentrations at the lower end of the experimental range may have influenced Fe absorption, tissue deposition, and retention through changes in physiological Fe homeostasis. Consequently, the RBV estimates should be interpreted as comparative measures of biological response among Fe sources under the dietary conditions tested, rather than as direct estimates applicable to diets formulated solely according to NRC Fe requirements.

## Conclusions

The present study demonstrated that the biological response to dietary Fe supplementation in growing Japanese quail depended on both Fe source and dietary concentration, although the magnitude and direction of these effects varied among biological endpoints. Growth performance was relatively insensitive for discriminating among Fe sources, whereas bone characteristics, tissue Fe deposition, and fecal Fe excretion provided more informative indicators of Fe utilization. Relative bioavailability estimates were strongly endpoint- and model-dependent and did not support a consistent ranking of Fe sources across all response criteria. Chelated-Fe produced favorable responses for several bone and tissue endpoints, while nano-Fe was associated with lower fecal Fe excretion than FeSO₄, a response consistent with greater apparent Fe retention or utilization but not sufficient to establish improved overall biological availability or safety. Fe oxide generally showed less favorable utilization characteristics. However, several RBV estimates were accompanied by wide and overlapping confidence intervals, limiting definitive conclusions regarding differences among sources based solely on point estimates. Collectively, these findings emphasize that Fe-source bioavailability should be interpreted using multiple complementary biological endpoints and appropriate dose–response models rather than a single RBV estimate. Because direct toxicological and long-term safety assessments were not conducted, the potential nutritional advantages of nano-Fe should also be interpreted cautiously.

## Author contributions statement

**Mehran Mehri** contributed substantially to the conception and design of the study, the acquisition of data (including performing experiments and collecting data), and the analysis and interpretation of data. He drafted the main manuscript text and critically revised it for important intellectual content. Mehran Mehri reviewed and approved the final version of the manuscript and agrees to be personally and publicly accountable for all aspects of the work, including investigating and resolving any questions regarding its accuracy or integrity. **Mahmoud Ghazaghi** and **Meysam Pourtaheri** contributed substantially to the acquisition of data by performing experiments and collecting data. They critically reviewed the manuscript for key intellectual content and provided valuable input during revision. Both reviewed and approved the final version of the manuscript and agree to be personally and publicly accountable for all aspects of the work, including investigating and resolving any questions regarding its accuracy or integrity. **Farzad Bagherzadeh-Kasmani, Mehravar Mehri, Hassan Saleh**, and **Morteza Asghari-Moghadam** contributed substantially to the interpretation of data and critically revised the manuscript for essential intellectual content. All four reviewed and approved the final version of the manuscript and agree to be personally and publicly accountable for all aspects of the work, including investigating and resolving any questions regarding its accuracy or integrity.

## Funding declaration

This research received no external funding.

## Disclosures

The authors declare that they have no known competing financial interests or personal relationships that could have appeared to influence the work reported in this paper.
